# Water Quality and Brain Function

**DOI:** 10.3390/ijerph15010002

**Published:** 2017-12-21

**Authors:** Stephen C. Bondy, Arezoo Campbell

**Affiliations:** 1 Center for Occupational and Environmental Health, Department of Medicine, University of California, Irvine, CA 92617-1830, USA; 2 Department of Pharmaceutical Sciences, Western University of Health Sciences, Pomona, CA 91766-1854, USA; acampbell@westernu.edu

**Keywords:** drinking water quality, reservoirs, neurotoxic metals, neurotoxic organics, public health, brain function

## Abstract

In the United States, regulations are in place to ensure the quality of drinking water. Such precautions are intended to safeguard the health of the population. However, regulatory guidelines may at times fail to achieve their purpose. This may be due to lack of sufficient data regarding the health hazards of chronic low dose exposure to contaminants or the introduction of new substances that pose a health hazard risk that has yet to be identified. In this review, examples of different sources of contaminants in drinking water will be discussed, followed by an evaluation of some select individual toxicants with known adverse neurological impact. The ability of mixtures to potentially cause additive, synergistic, or antagonistic neurotoxic responses will be briefly addressed. The last section of the review will provide examples of select mechanisms by which different classes of contaminants may lead to neurological impairments. The main objective of this review is to bring to light the importance of considering trace amounts of chemicals in the drinking water and potential brain abnormalities. There is continued need for toxicology studies to better understand negative consequences of trace amounts of toxins and although it is beyond the scope of this brief overview it is hoped that the review will underscore the paucity of studies focused on determining how long-term exposure to minute levels of contaminants in drinking water may pose a significant health hazard.

## 1. Introduction

The purity of drinking water forms a critical basis for overall considerations of public health since consumption involves the whole population. Although stringent regulations are in place to ensure that drinking water does not contain harmful chemicals, there are trace amounts of impurities. The overall quality of the drinking water is dependent on the source and purification processes used. Contamination of water supplies rarely entails acute evidence of poisoning but rather encompasses a gradual and progressive impairment of health. This is due to chronic low-dose exposure that leads to bioaccumulation of water-soluble amphiphilic environmental toxins that can also be concentrated in lipid deposits. Once a threshold concentration is reached, cellular dysfunction may ensue. Thus, exposure to trace amounts of water contaminants may pose a risk for increased incidence of slow progressing diseases that are generally associated with aging or impaired development. Many of these disorders involve the central nervous system (CNS), which is especially susceptible to damage during development and then with acceleration of neurodegenerative changes during aging. 

The adverse consequences of environmental contaminants that are present in drinking water are based on the developmental phase of organisms. For instance, in utero exposures that cause impaired mitochondrial function can lead to insufficient energy production. Such disruption of normal cellular activity may in turn interrupt proper CNS growth. Another early consequence of exposure to contaminants may be abnormalities in regulated gene expression necessary for appropriate development. Genetic imprinting may also occur during early CNS growth that do not immediately manifest changes in function. However, later in the lifespan of an individual, when aging processes work in concert with these early genetic modifications, predisposition to disease may be enhanced. Exposure during later stages of lifespan may compound age-associated deterioration of CNS performance. For instance, an increase in both oxidative stress and inflammation is observed in senescence. As reviewed in this article, many of the contaminants present in drinking water increase one or both of these processes, which are thought to be key contributors to neurodegenerative disorders. The focus of this review is mainly on adverse consequences in the adult brain rather than direct changes to the fetal CNS. 

## 2. Sources of Contamination of Drinking Water

### 2.1. Industrial Waste

Many industrial processes use large quantities of water, and the runoff from these has the potential to pollute residential water supplies. Chemicals reaching water supplies in this manner include both inorganic salts and organic compounds. Recently, hydraulic fracturing (fracking), a method used to extract shale gas, has had a major impact on the quality of water leaving sites of this activity. Following drilling, there is a potential that hydrocarbons such as methane, ethane, and propane may leak and contaminate shallow groundwater. Surface water contamination can also occur from the injected hydraulic fracturing fluid, which contains a variety of chemicals including acids, surfactants, acrylic polymers, and borate compounds, amongst other factors [1]. The presence of neurotoxic acrylamide in water supplies, due to breakdown of polyacrylamide grouting agents, is generally overshadowed by the much greater amounts found in cooked foods and coffee [2] and thus is not discussed here.

### 2.2. Agricultural Runoff

Runoff of materials applied to agricultural crops that often end up in water sources, include pesticide and fertilizer residues. Irrigation with polluted water containing a range of agrochemicals are dominant sources of metals and organic compounds in agricultural soils [3,4]. In addition, livestock farming can add animal wastes and bacteria to the water effluent from fields. Finally, run-off from garden chemicals can make a contribution to the water burden of contaminants. One example of an agricultural contaminant in water sources is nitrate. Nitrate is commonly found because of the prevalent use of nitrogen fertilizers [5]. Neural tube defects have been shown to be four times greater in the progeny of women whose public water supply contained nitrate above the US maximum contaminant level [6].

A large number of pesticides have neurotoxic properties [7]. These are often present in combinations with unknown synergistic or antagonistic consequences. Maximum water concentration levels (MCVs) and regulatory guidance levels (RGVs) of many pesticides vary by several orders of magnitude. Furthermore, these values often exceed the calculated upper values for human health risk allowing for uncertainty. Many implied dose limits are above tolerable daily intake values. Despite the fact that worldwide jurisdictions are making efforts to regulate pesticide levels in water and also in other sources, as yet, current worldwide regulatory mechanisms related to drinking water do not provide safe standards in a manner so as to protect public health. Much improvement in this area is clearly needed [8,9]. 

### 2.3. Water Treatment

Another threat to the quality of water stores ironically comes from procedures intended to improve the potability of water. Formation of a biofilm in drinking water distribution systems allows microbial growth [10]. One method of disinfection is to use of chlorine and its derivatives. Interaction of these agents with trace amounts of organic compounds in water can lead to formation of stable and potentially harmful chlorinated organics. Chlorinated wastewater has been shown to be cytotoxic to mammalian cells [11]. A combination of chlorine and ammonia, used for disinfecting water causes erosion of copper piping that may increase the content of the metal in the drinking water. The impact of chlorine on corroding copper pipes is pH dependent [12]. Another potentially harmful material added to water in order to precipitate organic matter and thus to clarify water involves the application of alumina as a means of coagulating particulate contaminants. This can increase the aluminum content of water [13]. The increasing prevalence of acid rain leads to aluminum leaching from rocks and can thus further elevate the aluminum burden of water resources. Epidemiological evidence reveals that aluminum levels in drinking water are related to the incidence of Alzheimer’s disease (AD) [14]. This is supported by laboratory data with experimental animals exposed to levels of aluminum in drinking water that parallel those found in some residential supplies [15,16]. However, the issue of a causal relation of Al in water and the promotion of AD remains controversial [17].

A recent study evaluated how exposure to disinfection by-products found in tap water during pregnancy relate to adverse neurodevelopmental consequences of the child. Although there was a positive correlation between exposure to disinfection by-products and mental score for girls at one year of age, the difference did not persist when the evaluation was conducted at 4–5 years of age [18]. Thus, more studies are needed to better understand if and how trace amounts of disinfectants in the tap water may have neurodevelopmental or neurodegenerative consequences. 

### 2.4. Water Conduits

Water delivery through metal-lined conduits can be another factor that can impair the quality of the final product emerging from household taps. While the use of lead piping has been greatly curtailed in recent years, lead soldering is still a popular means of annealing piping. The evidence that even low levels of lead is a developmental hazard to human populations is unambiguous [19]. In addition, the use of copper tubing is currently widespread. Newly emerging data involving both epidemiological reports and studies on experimental animals are increasingly indicating that water-borne copper can also be a source of neurotoxicity [20,21,22,23]. 

### 2.5. Consumer Products

Pharmaceutical agents can enter the aquatic environment and eventually the drinking water [24,25]. According to the Center for Disease Control and Prevention (CDC), 48.7% of the population uses at least one prescription drug every 30 days and some of these chemicals gradually get into the water sources. Water from public drinking water supply wells on Cape Cod frequently contained levels of the antibiotic sulfamethoxazole, the anticonvulsant phenytoin, and the surfactant perfluorooctane sulfonate at high levels [26]. 

In the aquatic environment, pharmacological agents may have adverse effects. Environmentally relevant doses of the anxiolytic drug oxazepam can alter the behavior of fish [27]. Thus, the presence of pharmacological agents in water supplies can lead to adverse consequences to aquatic life and eventually to human health. Metals can enter the water supply as a result of the improper disposal of lead containing batteries and of brake pads, which are high in zinc and copper. A number of metals such as cadmium, zinc, lead, and copper, present in brakes and tires, can also contaminate storm water runoff from roadways [28]. 

## 3. Consideration of Neurotoxic Potential of Individual Contaminants

### 3.1. Metals

#### 3.1.1. Lead (Pb)

The seepage of lead from industrial wastes into drinking water is an old problem. This is compounded by the existence of significant amounts of lead in some waters, especially those from low-lying areas [4,29]. Whereas lead toxicity was once believed to involve only a small population of industrially exposed workers, the neurotoxicity of very low levels of lead that can be relevant to large populations, has been widely recognized for the last 20 years. This adverse effect is especially pronounced in the developing fetal and neonatal brain. Low-dose exposure to lead has been associated with behavioral and cognitive impairments [30]. 

One of the more recent incidents involving lead exposure through the drinking water, is from Flint, Michigan. In an effort to cut costs, the city of Flint switched its water source from the Detroit Water and Sewage Department to Flint River water treated at Flint Water Service Center [31]. Many water samples tested subsequently contained lead above the safe level [32]. Blood lead levels in children were significantly increased [33]. In California, contamination from a lead battery recycling plant has led to community concerns regarding potential neurological impacts [34]. An analysis of blood lead levels in children less than six years old revealed that residential proximity to the plant had an impact on these levels [35].

The potential for lead to cause brain abnormalities is not limited to neurodevelopment. A hallmark of Alzheimer’s disease is senile plaques composed of amyloid beta (Aβ) protein derived from the amyloid precursor protein (APP). Prenatal lead exposure may have delayed long-term consequences in that it can influence Aβ-related biological pathways that have been implicated in Alzheimer’s disease [36]. Early gene imprinting by environmental lead exposure may subsequently enhance expression of genes associated with neurodegenerative disorders [37]. In monkeys, exposure to Pb during an early phase of brain development caused an increase in APP expression and amyloid deposition in later life [38,39]. 

#### 3.1.2. Aluminum (Al)

The prevalence of Al in drinking water varies considerably [40]. The metal is often added to water as a coagulant of organic matter and the residual levels that are inadvertently solubilized are considered innocuous. However, increasing evidence from both epidemiological and from laboratory studies, suggests that levels of Al found in some drinking water may be harmful. Chronic exposure to such levels can cause neuroinflammation and oxidative stress in experimental animals [15,21]. 

Al exposure may potentiate the progression of neurodegenerative disorders such as Alzheimer’s disease. This is strengthened by findings showing elevated Al in postmortem AD brain tissue [41,42,43]. Al is found in the cerebral arteries of AD patients [44] suggesting that the metal may disrupt aspects of the blood brain barrier [45]. Epidemiological studies have been able to strengthen this connection between Al ingestion and neurodegenerative disease. The prevalence of AD dose-dependently increased in areas where Al concentrations in the drinking water supply are high [46]. A meta-analysis of nine reports associated elevated Al concentrations with diminished cognitive performance [47]. A complicating factor in evaluation of the effects of Al in drinking water, is evidence from human and animal studies showing that the simultaneous presence of silicic acid appears to be protective against Al toxicity, presumably due to formation of an inert aluminosilicate [48,49]. It is difficult to link one specific exposure to AD since it is a pleiotropic disease with many causes. The positive and negative evidence for Al being a factor in AD has recently been evaluated and summarized [50].

#### 3.1.3. Copper (Cu)

Low concentrations of copper salts in water that were previously considered harmless may also be related to adverse neurological effects. Unlike lead and aluminum, Cu is a trace element that has a biological role as a cofactor in many enzymes. However, because the free forms of Cu are toxic, there are many mechanisms that are in place to keep the metal bound to proteins. Two hereditary disorders that disrupt Cu homeostasis (Menkes’ and Wilson’s diseases) illustrate the neurotoxic potential of Cu [51]. 

Cu in the drinking water has been shown to be more toxic than corresponding concentrations of the metal that are present in food [52]. The levels of the metal in drinking water are governed by the pH and the types of conduits that are used. Households that use Cu tubing in the plumbing system have higher concentrations of the metal due to gradual corrosion. However, the amounts of Cu in tap water are much lower if the water is allowed to run before use [53]. The establishment of regulations concerning copper content of residential water set the maximum standard for copper as 1.3 ppm [54]. Levels of up to 7.8 mg/L can be reached if water remains stagnant in corroding copper pipes [55].

Increased copper ingestion from drinking water can be associated with gastrointestinal symptoms [56]. There are concerns that the metal may also play a causative role in neurodegenerative disorders such as Alzheimer’s disease and Parkinson’s disease (PD). These concerns are based on the findings that the non-ceruloplasmin bound component of Cu is increased in the serum of AD patients [57] and cerebrospinal fluid of patients with PD [58]. There is evidence from a transgenic mouse model of AD that chronic copper exposure accelerates disease-associated pathology [22,23] and occupational exposure to Cu has been linked to an increased risk of PD [59]. Free Cu has been associated with cognitive decline [60] and this observation strengthens its potential role in AD [61].

Specific Cu-binding sites are present on the amyloid precursor protein [62] as well as the amyloid peptide [63] where the metal attaches [64]. Upon binding, Cu undergoes redox cycling which leads to formation of reactive oxygen intermediates [65,66]. Low-dose exposure to Cu in the drinking water significantly increased markers of oxidative stress in the brain of exposed animals [21] and increased activation of the transcription factor, AP-1 [67]. Since AP-1 is activated by redox status, the mechanism by which Cu modulates the transcription factor is likely related to its stimulation of oxidative processes. Inability of microglia to sequester Cu bound to amyloid plaques may enhance inflammatory events known to be exacerbated in AD [68]. Most recently, levels of Cu as low as 2 µM (a level only 10% of maximal levels of Cu recommended by the EPA) in the drinking water of aged mice, have been found to inhibit the actions of low-density lipoprotein receptor-related protein 1 (LRP1), which is involved in the transport of β-amyloid out of cells. This leads to accumulation of β-amyloid and neuroinflammation within the brain [69]. Overall, this concordance of laboratory and studies of human populations suggests a major, largely unacknowledged hazard.

Thus, Cu in drinking water may have the ability to cause adverse neurological effects through several separate but intertwined mechanisms. 

#### 3.1.4. Arsenic and Cadmium

The presence of inorganic arsenic or cadmium in drinking water can present a serious hazard. Chronic arsenic poisoning is found in large parts of Bangladesh and adjoining parts of India, where ground water is severely contaminated with heavy metals [70]. One study of nearly a million subjects in a part of West Bengal reported a prevalence rate of arsenicosis in over 15% of the inhabitants. The highest level of arsenic found in drinking water was over 1300 µg/L and values of over 100 µg/L were common. Peripheral neuropathy was present in 16% of cases [71]. 

Arsenic exposure has also been linked to neurodevelopmental abnormalities. For example, in a case control study conducted in Bangladesh, mothers with higher arsenic exposure and folate deficiency were found to be at a higher risk of giving birth to an infant with neural tube defects. This was associated with histone modifications, which were taken to imply epigenetic effects [72]. Furthermore, cognitive [73,74] and motor [75] function is lower in children that consume arsenic contaminated water for a prolonged period. This metal may also enhance biological processes associated with neurodegenerative disorders. For example, arsenic promotes accumulation of α-synuclein, a neuronal protein that plays an important role in Parkinson’s disease [76]. An epidemiological relation between arsenic exposure and a higher incidence of neurodegenerative disorders has been reported [77]. This may be attributable to the ability of arsenic to promote oxidative stress and inflammation, both of which are associated with neurodegeneration [78].

The hazards of arsenic in drinking water are not confined to third world countries. Some parts of the US, notably Texas and the Great Lakes Basin, have arsenic levels of over 50 µg/L in groundwater [79]. 

While the neurotoxic aspects of arsenic are overshadowed by its potential as a carcinogen, damage to the developing nervous system poses a grave long-term risk. Similarly, while cadmium exposure is associated with nephrotoxicity, low doses of cadmium in drinking water can promote excess free radical related oxidative events. The brain appears to be more sensitive to such changes than other organs [80]. It is likely that exposure to neonates is more harmful compared to adults [81]. Cadmium levels in drinking water have been found to exceed the permissible limits of the World Health Organization in both Egypt and Iran [82,83]. The hazard posed by seemingly low-level exposures to several heavy metals, such as cadmium, mercury and lead is exacerbated by the tendency of these metals to accumulate in tissues over time. 

### 3.2. Organic Materials

#### 3.2.1. Halogenated Residues

Organochlorine pesticides owe their effectiveness to their stability rather than to their reactivity. This durability allows prolonged allosteric interaction with key receptor sites and ion channels. Their non-reactiveness is the same quality that can permit their persistence in aqueous media. Another class of stable organohalogen compounds that has diffused into the environment, includes polychlorinated biphenyls (used as electrical insulators) and polybrominated biphenyls (used as fire retardants). The inadvertently produced dioxins are also in this group. Due to their exceptionally low reactivity, trace amounts of these materials can persist for extended periods in water supplies. Despite their inertness, such compounds can selectively target specific biological sites and impede their function for long periods by allosteric means. Their neurotoxicity is well established [84].

A further presence of organohalogens in drinking water comes from the use of chlorine, bromine or their derivatives such as chloramines and chlorine dioxide as a means of water disinfection. Water sterilization byproducts include trihalomethanes, haloacetic acids, haloacetaldehydes, haloacetonitriles, haloamines, nitrosamines, and halobenzoquinones [85]. While most of these have not been extensively studied, the neurotoxicity of several disinfection byproducts, including dibromacetic acid found in water stocks, has been described [86]. 

An association between extended exposure to trichloroethylene (TCE) and Parkinson’s disease has been reported: the exposure of animals to TCE can lead to striatal degeneration and the onset of parkinsonian characteristics. This has been attributed to mitochondrial dysfunction perhaps due to the formation of chloral from TCE and the subsequent synthesis of 1-trichloromethyl-1,2,3,4-tetrahydro-beta-carboline (TaClo) a specific dopaminergic neurotoxin [87]. 

#### 3.2.2. Acrylamide

Polyacrylamide is a grouting agent and is also an effective flocculent in water clarification. It is useful in lining pipes, wells, and canals in order to reduce loss of water by leakage. Polyacrylamide is also present in herbicide blends in order to increase viscosity. The degradation of polyacrylamide to the monomeric acrylamide can present a toxic hazard to water supplies. There have been cases of poisoning following consumption of water from recently grouted pipes or wells. These have led to severe neurotoxic effects, including ataxia, hallucinations and memory disturbances [88].

There are several reasons why acrylamide contamination is generally not a major issue. Acrylamide is biodegraded in water, with a half-life of around two days, and it is not bioaccumulated. Also, the acrylamide intake from commonly eaten food is several orders of magnitude greater than that to be expected from water [89]. 

#### 3.2.3. Bisphenol A

Bisphenol A (BPA) is an important chemical used in large amounts in the production of various plastics including polycarbonates and epoxy resins. In addition to being used in manufacture of bottles and linings of cans containing food products, epoxy resins are also used to line water supply pipes. By acting on estrogen receptors, bisphenol A is a developmental toxin and a carcinogen. It also causes neuronal apoptosis together with metabolic and behavioral changes by mechanisms not involving the estrogen receptor [90]. The neurotoxicity of BPA is controversial since another study (supported by the polycarbonate industry) failed to find evidence of neurotoxicity [91]. While the BPA content of bottled drinking water is a major concern, this chemical has also been found in tap water in several countries, including the US, at levels, that can be greater than that of bottled water [92].

#### 3.2.4. Other Organic Contaminants of Anthropogenic Origin

Innocuous materials such as synthetic musks (for example, 1,3,4,6,7,8-hexahydro-4,6,6,7,8,8-hexamethylcyclopenta[γ]-2-benzopyran) are widely used in the perfume industry. They have been reported to contaminate water supplies in China. Since these are very stable compounds, with very low biodegradability, they are capable of bioaccumulation and thus their potential hazard needs to be carefully evaluated [93,94]. Other agents which find widespread use in personal care products, include surfactants such as 4-nonylphenol. These are also found in urban stream water [94]. 4-nonylphenol is known to be neurotoxic and to activate retinoid receptors [95].

### 3.3. Mixtures of Contaminants

While a variety of agents may individually be within the safety levels set by regulatory agencies, mixtures of contaminants may produce a significant overall health hazard. In view of all potential interrelationships between toxicants, the health consequences of such multi-component constituents are hard to predict. For example, tap water has been linked to neural tube defects in mice embryos, and since no single contaminant could be linked to the observation, the authors suggest that the effects may be caused by the combinations of low-dose contaminants [96]. Concurrent exposure to a range of toxic heavy metals, including lead, cadmium, arsenic and methylmercury are of notable concern in view of their persistent effects on the brain. The exact toxicological mechanisms invoked by exposure to such mixtures are still unclear, however they influence many common metabolic pathways that are related to cognitive dysfunction [97]. A further consequence of the presence of several agents in water impacting on related processes is that additive, synergistic, or antagonistic interactions between water contaminants are likely. The complexity of this issue is illustrated by a study of four toxic metals in an isolated cell system. With increasing metal concentration, effects were additive, then synergistic, and finally antagonistic [98]. It is significant that at the lower concentrations studied, which are most likely to reflect the real-life situation, effects were additive or synergistic. Similar additive or synergistic interactions have been found in exposures of intact animals to levels of lead, cadmium, and arsenic that reflect Lowest Observed Effect Levels (LOELs) of each component [99]. There is evidence from neurodevelopmental studies in Bangladesh that arsenic, lead, and manganese can potentiate one another’s toxicity. In human populations, lead and manganese appear to interact in a non-additive synergistic manner [100]. Synergistic neurotoxic interactions between metals at levels paralleling those found in some Indian ground waters have also been reported in a more defined animal model system [101].

The question thus arises as to whether the regulatory standards for individual compounds are in fact adequate for the real-world situation where the water content of a single constituent cannot be considered in isolation. This is obviously a complex and difficult issue to address but is relevant to water supplies which are rarely affected by a solitary contaminant. 

## 4. Mechanisms of Neurotoxicity

**Figure 1:**
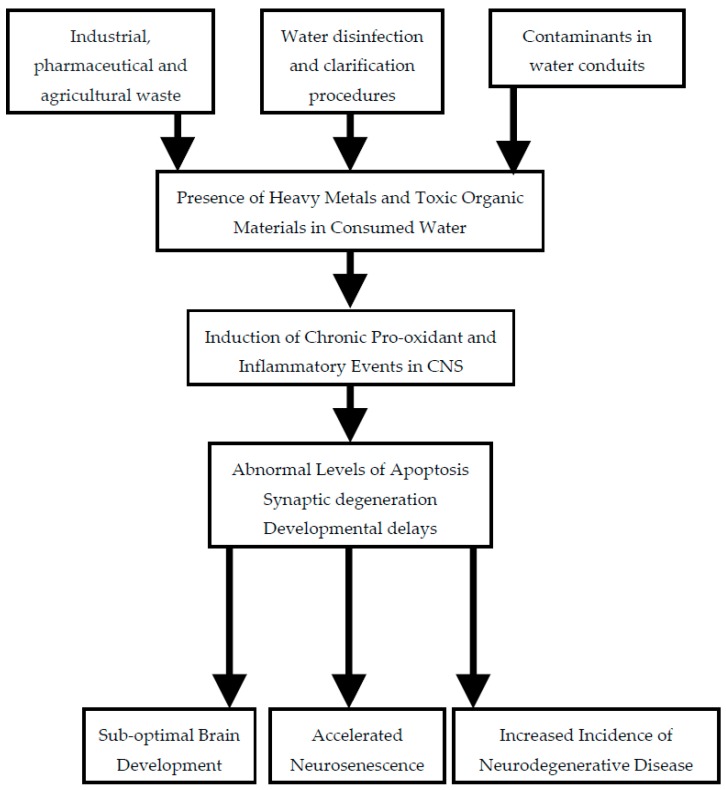
Sources of neurotoxic agents in drinking water and their potential consequences. CNS: central nervous system.

While each of the agents potentially contaminating water supplies discussed above has a characteristic profile of neurotoxicity, there are some effects that are common to many chemicals. This is not surprising, as these events frequently constitute signs of an unhealthy cell with sub-optimal metabolic activity. One such process is an enhancement of inflammatory pathways consequent to activation of innate immune responses. Another frequent occurrence is the presence of excess levels of pro-oxidant activity. Both are common mechanisms associated with neurodegenerative diseases. The pathways by which quality of drinking water may be compromised and thus impact on brain health are summarized in Figure 1.

### 4.1. Neuroinflammation 

The immune system is composed of a system of cellular and molecular mediators that orchestrate resistance against various insults, including protection against chemicals, which may harm the body. Innate immunity is a first line of defense that uses the inflammatory response to immediately recruit cells to guard against potentially damaging substances. Key participants in inflammation are cytokines and chemokines, which work together in a protective manner [102]. Since the blood brain barrier provides protection for the brain, it was previously thought that the CNS is immunologically privileged. However, it has been shown that this barrier does not confer immune privilege on the brain and that there is active interaction of the CNS with peripheral immune cells [103]. Furthermore, the brain is capable of innate immune activation following environmental insults [15]. Although acute immune responses in the brain that eventually resolve are protective, extended immune activation can occur following a transient toxic insult and this can lead to neurodegeneration [104]. 

In AD, chronic neuroinflammation is enhanced with disease progression and is considered to be one of the major mechanisms of disease pathology [105,106]. The most important inflammatory cytokines are tumor necrosis factor alpha (TNF-α) and interleukins 1 & 6 (IL-1, IL-6), and levels of these are elevated in AD brains [107]. Inflammatory cytokines can disrupt the integrity of the blood brain barrier resulting in access of environmental toxicants into the brain parenchyma [108]. 

### 4.2. Oxidative Stress

Reactive oxygen species have a physiological role, and the respiratory burst is a mechanism by which immune cells destroy pathogens [102]. However, abnormal or persistent levels of such oxidant species can be damaging. Contaminants such as redox active metals, once accumulated, may cause neurotoxicity by promotion of free radical formation.

Effective mitochondrial functioning is necessary for maintaining CNS health. This organelle provides energy to actively transport nutrients into the brain and export environmental neurotoxins that may have inadvertently entered the brain. Drinking water contaminants that disrupt mitochondrial function or enhance production of reactive oxygen species may in time exhaust defensive antioxidant processes. The ensuing surge in oxidative stress can lead to abnormal brain function. Indeed, mitochondrial dysfunction and oxidative stress are commonly observed in patients with neurodegenerative disorders such as Alzheimer’s disease [109,110,111,112]. 

## 5. Conclusions

There have been major improvements regarding the quality of residential water supplies. However, there is still considerable variance in different communities, in part due to differing standards. In addition to the World Health Organization and federal government, each state in the US has its individual standards. A unified set of guidelines for drinking water, based on scientific evidence and economic feasibility, would effectively protect global health.

One approach to mitigating the adverse consequences of contaminants in drinking water is to institute green chemistry practices. For instance, the green pharmacy movement is a program aimed at designing drugs that are more biodegradable or improving wastewater treatment by processes such as ozonation, which can further degrade pharmaceutical agents [24,113,114]. Another proposed approach is to enhance planning and technology. An example of this approach would be source separation of wastewater so that “greywater” that is derived from showers or washing machines is collected separately from “blackwater” that is derived from the toilet. The separated wastewater can then be treated more efficiently [115]. Such global developments in technology would limit contaminations and ensure that there will be sufficient clean water to sustain a rapidly developing world population.

## References

[1] Vengosh A., Jackson R.B., Warner N., Darrah T.H., Kondash A. (2014). A critical review of the risks to water resources from unconventional shale gas development and hydraulic fracturing in the United States. Environ. Sci. Technol..

[2] Pérez H.L., Osterman-Golkar S. (2003). A sensitive gas chromatographic-tandem mass spectrometric method for detection of alkylating agents in water: Application to acrylamide in drinking water, coffee and snuff. Analyst.

[3] Zhang L., Mo Z., Qin J., Li Q., Wei Y., Ma S., Xiong Y., Liang G., Qing L., Chen Z. (2015). Change of water sources reduces health risks from heavy metals via ingestion of water, soil, and rice in a riverine area, South China. Sci. Total Environ..

[4] Islam M.A., Romić D., Akber M.A., Romić M. (2017). Trace metals accumulation in soil irrigated with polluted water and assessment of human health risk from vegetable consumption in Bangladesh. Environ. Geochem. Health.

[5] Brender J.D., Weyer P.J. (2016). Agricultural compounds in water and birth defects. Curr. Environ. Health Rep..

[6] Croen L.A., Todoroff K., Shaw G.M. (2001). Maternal exposure to nitrate from drinking water and diet and risk for neural tube defects. Am. J. Epidemol..

[7] Costa L.G., Giordano G., Guizzetti M., Vitalone A. (2008). Neurotoxicity of pesticides: A brief review. Front. Biosci..

[8] Li Z., Jennings A. (2017). Worldwide regulations of standard values of pesticides for human health risk control: A review. Int. J. Environ. Res. Public Health.

[9] Li Z., Jennings A.A. (2017). Implied maximum dose analysis of standard values of 25 pesticides based on major human exposure pathways. AIMS Public Health.

[10] Liu S., Gunawan C., Barraud N., Rice S.A., Harry E.J., Amal R. (2016). Understanding, monitoring, and controlling biofilm growth in drinking water distribution systems. Environ. Sci. Technol..

[11] Du Y., Wu Q.Y., Lu Y., Hu H.Y., Yang Y., Liu R., Liu F. (2017). Increase of cytotoxicity during wastewater chlorination: Impact factors and surrogates. J. Hazard. Mater..

[12] Lytle D.A., Liggett J. (2016). Impact of water quality on chlorine demand of corroding copper. Water Res..

[13] Gopal K., Srivastava S.B., Shukla S., Bersillon J.L. (2004). Contaminants in drinking water and its mitigation using suitable adsorbents: An overview. J. Environ. Biol..

[14] Rondeau V., Jacqmin-Gadda H., Commenges D., Helmer C., Dartigues J.F. (2009). Aluminum and silica in drinking water and the risk of Alzheimer’s disease or cognitive decline: Findings from 15-year follow-up of the PAQUID cohort. Am. J. Epidemiol..

[15] Campbell A., Becaria A., Lahiri D.K., Sharman K., Bondy S.C. (2004). Chronic exposure to aluminum in drinking water increases inflammatory parameters selectively in the brain. J. Neurosci. Res..

[16] Bondy S.C. (2016). Low levels of aluminum can lead to behavioral and morphological changes associated with Alzheimer’s disease and age-related neurodegeneration. Neurotoxicology.

[17] Santibáñez M., Bolumar F., García A.M. (2007). Occupational risk factors in Alzheimer’s disease: A review assessing the quality of published epidemiological studies. Occup. Environ. Med..

[18] Villanueva C.M., Gracia-Lavedan E., Julvez J., Santa-Marina L., Lertxundi N., Ibarluzea J., Llop S., Ballester F., Fernández-Somoano A., Tardón A. (2018). Drinking water disinfection by-products during pregnancy and child neuropsychological development in the INMA Spanish cohort study. Environ. Int..

[19] Liu J., Gao D., Chen Y., Jing J., Hu Q., Chen Y. (2014). Lead exposure at each stage of pregnancy and neurobehavioral development of neonates. Neurotoxicology.

[20] Sparks D.L., Schreurs B. (2003). Trace amounts of copper in water induce beta-amyloid plaques and learning deficits in a rabbit model of Alzheimer’s disease. Proc. Natl. Acad. Sci. USA.

[21] Becaria A., Lahiri D.K., Bondy S.C., Chen D., Hamadeh A., Li H., Taylor R., Campbell A. (2006). Aluminum and copper in drinking water enhance inflammatory or oxidative events specifically in the brain. J. Neuroimmunol..

[22] Kitazawa M., Cheng D., Laferla F.M. (2009). Chronic copper exposure exacerbates both amyloid and tau pathology and selectively dysregulates cdk5 in a mouse model of AD. J. Neurochem..

[23] Kitazawa M., Hsu H.W., Medeiros R. (2016). Copper exposure perturbs brain inflammatory responses and impairs clearance of amyloid-beta. Toxicol. Sci..

[24] Blair B.D. (2016). Potential upstream strategies for the mitigation of pharmaceuticals in the aquatic environment: A brief review. Curr. Environ. Health Rep..

[25] Brezina E., Prasse C., Meyer J., Muckter H., Ternes T.A. (2017). Investigation and risk evaluation of the occurrence of carbamazepine, oxcarbazepine, their human metabolites and transformation products in the urban water cycle. Environ. Pollut..

[26] Schaider L.A., Rudel R.A., Ackerman J.M., Dunagan S.C., Brody J.G. (2014). Pharmaceuticals, perfluorosurfactants, and other organic wastewater compounds in public drinking water wells in a shallow sand and gravel aquifer. Sci. Total Environ..

[27] Brodin T., Fisk J., Josnsson M., Klaminder J. (2013). Dilute concentrations of a psychiatric drug alter behavior of fish from natural populations. Science.

[28] McKenzie E.R., Money J.E., Green P.G., Young T.M. (2009). Metals associated with stormwater-relevant brake and tire samples. Sci. Total Environ..

[29] Choudhury D., Gupta S. (2017). Impact of waste dump on surface water quality and aquatic insect diversity of Deepor Beel (Ramsar site), Assam, North-east India. Environ. Monit. Assess..

[30] Finkelstein Y., Markowitz M.E., Rosen J.F. (1998). Low-level lead-induced neurotoxicity in children: An update on central nervous system effects. Brain Res. Rev..

[31] Zahran S., McElmurry S.P., Sadler R.C. (2017). Four phases of the Flint Water Crisis: Evidence from blood lead levels in children. Environ. Res..

[32] Edwards M. (2015). Our Sampling of 252 Homes Demonstrates a High Lead in Water Risk: Flint Should Be Failing to Meet the EPA Lead and Copper Rule. http://flintwaterstudy.org/2015/09/our-sampling-of-252-homes-demonstrates-a-high-lead-in-water-risk-flint-should-be-failing-to-meet-the-epa-lead-and-copper-rule.

[33] Hanna-Attisha M., LaChance J., Sadler R.C., Schnepp A.C. (2016). Elevated blood lead levels in children associated with the flint drinking water crisis: A spatial analysis of risk and public health response. Am. J. Public Health.

[34] Barboza T., Poston B. (2016). Brain-Damaging Lead Levels near Battery Plant Found as High as 100 Times above Health Limits. http://www.latimes.com/local/california/la-me-ln-dangerous-lead-levels-20160714-snap-story.html.

[35] Department of Toxic Substances Control (DTSC) (2016). An Analysis of Children’s Blood Lead Levels in the Area around the Exide Site. http://dtsc.ca.gov/HazardousWaste/Projects/upload/An-Analysis-of-Children-s-Blood-Lead-Levels-in-the-Area-Around-the-Exide-Site.pdf.

[36] Mazumdar M., Xia W., Hofmann O., Gregas M., Ho Sui S., Hide W., Yang T., Needleman H.L., Bellinger D.C. (2012). Prenatal lead levels, plasma amyloid β levels, and gene expression in young adulthood. Environ. Health Perspect..

[37] Lahiri D.K., Maloney B. (2010). The “LEARn” (Latent Early-life Associated Regulation) model integrates environmental risk factors and the developmental basis of Alzheimer’s disease, and proposes remedial steps. Exp. Gerontol..

[38] Basha M.R., Wei W., Bakheet S.A., Benitez N., Siddiqi H.K., Ge Y.W., Lahiri D.K., Zawia N.H. (2005). The fetal basis of amyloidogenesis: Exposure to lead and latent overexpression of amyloid precursor protein and beta-amyloid in the aging brain. J. Neurosci..

[39] Wu J., Basha M.R., Brock B., Cox D.P., Cardozo-Pelaez F., McPherson C.A., Harry J., Rice D.C., Maloney B., Chen D. (2008). Alzheimer’s disease (AD)-like pathology in aged monkeys after infantile exposure to environmental metal lead (Pb): Evidence for a developmental origin and environmental link for AD. J. Neurosci..

[40] World Health Organization (1996). Guidelines for Drinking-Water Quality, Second Edition. Volume 2: Health Criteria and Other Supporting Information.

[41] Perl D.P., Brody A.R. (1980). Alzheimer’s disease: X-ray spectrometric evidence of aluminum accumulation in neurofibrillary tangle-bearing neurons. Science.

[42] Xu N., Majidi V., Markesbery W.R., Ehmann W.D. (1992). Brain aluminum in Alzheimer’s disease using an improved GFAAS method. Neurotoxicology.

[43] Mirza A., King A., Troakes C., Exley C. (2017). Aluminium in brain tissue in familial Alzheimer’s disease. J. Trace Elem. Med. Biol..

[44] Bhattacharjee S., Zhao Y., Hill J.M., Culicchia F., Kruck T.P., Percy M.E., Pogue A.I., Walton J.R., Lukiw W.J. (2013). Selective accumulation of aluminum in cerebral arteries in Alzheimer’s disease (AD). J. Inorg. Biochem..

[45] Guerriero F., Sgarlata C., Francis M., Maurizi N., Faragli A., Perna S., Rondanelli M., Rollone M., Ricevuti G. (2017). Neuroinflammation, immune system and Alzheimer disease: Searching for the missing link. Aging Clin. Exp. Res..

[46] McLachlan D.R., Bergeron C., Smith J.E., Boomer D., Rifat S.L. (1996). Risk for neuropathologically confirmed Alzheimer’s disease and residual aluminum in municipal drinking water employing weighted residential histories. Neurology.

[47] Meyer-Baron M., Schäper M., Knapp G., van Thriel C. (2007). Occupational aluminum exposure: Evidence in support of its neurobehavioral impact. Neurotoxicology.

[48] Gillette-Guyonnet S., Andrieu S., Nourhashemi F., de La Guéronnière V., Grandjean H., Vellas B. (2005). Cognitive impairment and composition of drinking water in women: Findings of the EPIDOS Study. Am. J. Clin. Nutr..

[49] Foglio E., Buffoli B., Exley C., Rezzani R., Rodella L.F. (2012). Regular consumption of a silicic acid-rich water prevents aluminium-induced alterations of nitrergic neurons in mouse brain: Histochemical and immunohistochemical studies. Histol. Histopathol..

[50] Bondy S.C., Campbell A., Aschner A., Costa L.G. (2017). Aluminum and neurodegenerative diseases. Advances in Neurotoxicology.

[51] Campbell A. (2006). The role of aluminum and copper on neuroinflammation and Alzheimer’s disease. J. Alzheimers Dis..

[52] Hébert C.D., Elwell M.R., Travlos G.S., Fitz C.J., Bucher J.R. (1993). Subchronic toxicity of cupric sulfate administered in drinking water and feed to rats and mice. Fundam. Appl. Toxicol..

[53] World Health Organization (2004). Copper in Drinking-Water: Background Document for Development of WHO Guidelines for Drinking Water Quality. http://www.who.int/water_sanitation_health/dwq/chemicals/copper.pdf.

[54] Environmental Protection Agency (EPA) Lead and Copper Rule. https://www.epa.gov/dwreginfo/lead-and-copper-rule.

[55] Spitalny K.C., Brondum J., Vogt R.L., Sargent H.E., Kappel S. (1984). Drinking-water-induced copper intoxication in a Vermont family. Pediatrics.

[56] Araya M., Olivares M., Pizarro F., González M., Speisky H., Uauy R. (2003). Gastrointestinal symptoms and blood indicators of copper load in apparently healthy adults undergoing controlled copper exposure. Am. J. Clin. Nutr..

[57] Squitti R., Pasqualetti P., Dal Forno G., Moffa F., Cassetta E., Lupoi D., Vernieri F., Rossi L., Baldassini M., Rossini P.M. (2005). Excess of serum copper not related to ceruloplasmin in Alzheimer disease. Neurology.

[58] Hozumi I., Hasegawa T., Honda A., Ozawa K., Hayashi Y., Hashimoto K., Yamada M., Koumura A., Sakurai T., Kimura A. (2011). Patterns of levels of biological metals in CSF differ among neurodegenerative diseases. J. Neurol. Sci..

[59] Gorell J.M., Johnson C.C., Rybicki B.A., Peterson E.L., Kortsha G.X., Brown G.G., Richardson R.J. (1999). Occupational exposure to manganese, copper, lead, iron, mercury and zinc and the risk of Parkinson’s disease. Neurotoxicology.

[60] Salustri C., Barbati G., Ghidoni R., Quintiliani L., Ciappina S., Binetti G., Squitti R. (2010). Is cognitive function linked to serum free copper levels? A cohort study in a normal population. Clin. Neurophysiol..

[61] Brewer G.J. (2012). Copper toxicity in Alzheimer’s disease: Cognitive loss from ingestion of inorganic copper. J. Trace Elem. Med. Biol..

[62] Barnham K.J., McKinstry W.J., Multhaup G., Galatis D., Morton C.J., Curtain C.C., Williamson N.A., White A.R., Hinds M.G., Norton R.S. (2003). Structure of the Alzheimer’s disease amyloid precursor protein copper binding domain. J. Biol. Chem..

[63] Dong J., Atwood C.S., Anderson V.E., Siedlak S.L., Smith M.A., Perry G., Carey P.R. (2003). Metal binding and oxidation of amyloid-beta within isolated senile plaque cores: Raman microscopic evidence. Biochemistry.

[64] Miller L.M., Wang Q., Telivala T.P., Smith R.J., Lanzirotti A., Miklossy J. (2006). Synchrotron-based infrared and X-ray imaging shows focalized accumulation of Cu and Zn co-localized with beta-amyloid deposits in Alzheimer’s disease. J. Struct. Biol..

[65] Dikalov S.I., Vitek M.P., Mason R.P. (2004). Cupric-amyloid beta peptide complex stimulates oxidation of ascorbate and generation of hydroxyl radical. Free Radic. Med. Biol..

[66] Guilloreau L., Combalbert S., Sournia-Saquet A., Mazarguil H., Faller P. (2007). Redox chemistry of copper-amyloid-beta: The generation of hydroxyl radical in the presence of ascorbate is linked to redox-potentials and aggregation state. Chem. Biochem..

[67] Lung S., Li H., Bondy S.C., Campbell A. (2015). Low concentrations of copper in drinking water increase AP-1 binding in the brain. Toxicol. Ind. Health.

[68] Zheng Z., White C., Lee J., Peterson T.S., Bush A.I., Sun G.Y., Weisman A., Petris M.J. (2010). Altered microglial copper homeostasis in a mouse model of Alzheimer’s disease. J. Neurochem..

[69] Singh I., Sagare A.P., Coma M., Perlmutter D., Gelein R., Bell R.D., Deane R.J., Zhong E., Parisi M., Ciszewski J. (2013). Low levels of copper disrupt brain amyloid-β homeostasis by altering its production and clearance. Proc. Natl. Acad. Sci. USA.

[70] Frisbie S.H., Ortega R., Maynard D.M., Sarkar B. (2002). The concentrations of arsenic and other toxic elements in Bangladesh’s drinking water. Environ. Health Perspect..

[71] Mazumder D.N., Ghosh A., Majumdar K.K., Ghosh N., Saha C., Mazumder R.N. (2010). Arsenic contamination of ground water and its health impact on population of district of Nadia, West Bengal, India. Indian J. Community Med..

[72] Tauheed J., Sanchez-Guerra M., Lee J.J., Paul L., Ibne Hasan M.O.S., Quamruzzaman Q., Selhub J., Wright R.O., Christiani D.C., Coull B.A. (2017). Associations between post translational histone modifications, myelomeningocele risk, environmental arsenic exposure, and folate deficiency among participants in a case control study in Bangladesh. Epigenetics.

[73] Tsai S.Y., Chou H.Y., The H.W., Chen C.M., Chen C.J. (2003). The effects of chronic arsenic exposure from drinking water on the neurobehavioral development in adolescence. Neurotoxicology.

[74] Wasserman G.A., Liu X., Parvez F., Ahsan H., Factor-Litvak P., Kline J., van Geen A., Slavkovich V., Loiacono N.J., Levy D. (2007). Water arsenic exposure and intellectual function in 6-year-old children in Araihazar, Bangladesh. Environ. Health Perspect..

[75] Parvez F., Wasserman G.A., Factor-Litvak P., Liu X., Slavkovich V., Siddique A.B., Sultana R., Sultana R., Islam T., Levy D. (2011). Arsenic exposure and motor function among children in Bangladesh. Environ. Health Perspect..

[76] Cholanians A.B., Phan A.V., Ditzel E.J., Camenisch T.D., Lau S.S., Monks T.J. (2016). From the Cover: Arsenic Induces Accumulation of α-Synuclein: Implications for Synucleinopathies and Neurodegeneration. Toxicol. Sci..

[77] Dani S.U. (2010). Arsenic for the fool: An exponential connection. Sci. Total Environ..

[78] Escudero-Lourdes C. (2016). Toxicity mechanisms of arsenic that are shared with neurodegenerative diseases and cognitive impairment: Role of oxidative stress and inflammatory responses. Neurotoxicology.

[79] US Geological Service Arsenic in Groundwater of the United States. http://water.usgs.gov/nawqa/trace/arsenic/index.html.

[80] Agnihotri S.K., Agrawal U., Ghosh I. (2015). Brain most susceptible to cadmium induced oxidative stress in mice. J. Trace Elem. Med. Biol..

[81] Stolakis V., Tsakiris S., Kalafatakis K., Zarros A., Skandali N., Gkanti V., Kyriakaki A., Liapi C. (2013). Developmental neurotoxicity of cadmium on enzyme activities of crucial offspring rat brain regions. Biometals.

[82] Mandour R.A., Azab Y.A. (2011). The prospective toxic effects of some heavy metals overload in surface drinking water of Dakahlia Governorate, Egypt. Int. J. Occup. Environ. Med..

[83] Fakhri F., Jafarzadeh S., Moradi B., Zandsalimi Y., Langarizadeh G., Amirhajeloo L.R., Mirzaei M. (2015). The non-carcinogenic risk of cadmium in bottled water in different age groups humans: Bandar Abbas City, Iran. Mater. Sociomed..

[84] Needham L.L., Barr D.B., Caudill S.P., Pirkle J.L., Turner W.E., Osterloh J., Jones R.L., Sampson E.J. (2005). ConIcentrations of environmental chemicals associated with neurodevelopmental effects in U.S. population. Neurotoxicology.

[85] Manasfi T., Coulomb B., Boudenne J.L. (2017). Occurrence, origin, and toxicity of disinfection byproducts in chlorinated swimming pools: An overview. Int. J. Hyg. Environ. Health.

[86] Moser V.C., Phillips P.M., Levine A.B., McDaniel K.L., Sills R.C., Jortner B.S., Butt M.T. (2004). Neurotoxicity produced by dibromoacetic acid in drinking water of rats. Toxicol. Sci..

[87] Zaheer F., Slevin J.T. (2011). Trichloroethylene and Parkinson disease. Neurol. Clin..

[88] Igisu H., Goto I., Kawamura Y., Kato M., Izumi K. (1975). Acrylamide encephaloneuropathy due to well water pollution. J. Neurol. Neurosurg. Psychiatry.

[89] Gökmen V. (2015). Acrylamide in Food: Analysis, Content and Potential Health Effects.

[90] Lee Y.M., Seong M.J., Lee J.W., Lee Y.K., Kim T.M., Nam S.Y., Kim D.J., Yun Y.W., Kim T.S., Han S.Y. (2007). Estrogen receptor independent neurotoxic mechanism of bisphenol A, an environmental estrogen. J. Vet. Sci..

[91] Stump D.G., Beck M.J., Radovsky A., Garman R.H., Freshwater L.L., Sheets L.P., Marty M.S., Waechter J.M., Dimond S.S., Van Miller J.P. (2010). Developmental neurotoxicity study of dietary bisphenol A in Sprague-Dawley rats. Toxicol. Sci..

[92] Karalius V.P., Harbison J.E., Plange-Rhule J., van Breemen R.B., Li G., Huang K., Durazo-Arvizu R.A., Mora N., Dugas L.R., Vail L. (2014). Bisphenol A (BPA) found in humans and water in three geographic regions with distinctly different levels of economic development. Environ. Health Insights.

[93] Zhang X., Xu Q., Man S., Zeng X., Yu Y., Pang Y., Sheng G., Fu J. (2013). Tissue concentrations, bioaccumulation, and biomagnification of synthetic musks in freshwater fish from Taihu Lake, China. Environ. Sci. Pollut. Res. Int..

[94] Peng F.J., Pan C.G., Zhang M., Zhang N.S., Windfeld R., Salvito D., Selck H., Van den Brink P.J., Ying G.G. (2107). Occurrence and ecological risk assessment of emerging organic chemicals in urban rivers: Guangzhou as a case study in China. Sci. Total Environ..

[95] Litwa E., Rzemieniec J., Wnuk A., Lason W., Krzeptowski W., Kajta M. (2014). Apoptotic and neurotoxic actions of 4-para-nonylphenol are accompanied by activation of retinoid X receptor and impairment of classical estrogen receptor signaling. J. Steroid Biochem. Mol. Biol..

[96] Mallela M.K., Were S.R., Hrubec T.C. (2011). Neural tube defects in mice exposed to tap water. Environ. Toxicol..

[97] Karri V., Schuhmacher M., Kumar V. (2016). Heavy metals (Pb, Cd, As and MeHg) as risk factors for cognitive dysfunction: A general review of metal mixture mechanism in brain. Environ. Toxicol. Pharmacol..

[98] Bae D.S., Gennings C., Carter W.H., Yang R.S., Campain J.A. (2001). Toxicological interactions among arsenic, cadmium, chromium, and lead in human keratinocytes. Toxicol. Sci..

[99] Whittaker M.H., Wang G., Chen X.Q., Lipsky M., Smith D., Gwiazda R., Fowler B.A. (2011). Exposure to Pb, Cd, and As mixtures potentiates the production of oxidative stress precursors: 30-day, 90-day, and 180-day drinking water studies in rats. Toxicol. Appl. Pharmacol..

[100] Valeri L., Mazumdar M.M., Bobb J.F., Henn B.C., Rodrigues E., Sharif O.I.A., Kile M.L., Quamruzzaman Q., Afroz S., Golam M. (2017). The joint effect of prenatal exposure to metal mixtures on neurodevelopmental outcomes at 20–40 months of age: Evidence from rural Bangladesh. Environ. Health Perspect..

[101] Ashok A., Rai N.K., Tripathi S., Bandyopadhyay S. (2015). Exposure to As-, Cd-, and Pb-mixture induces Aβ, amyloidogenic APP processing and cognitive impairments via oxidative stress-dependent neuroinflammation in young rats. Toxicol. Sci..

[102] Parham P. (2014). The Immune System.

[103] Carson M.J., Doose J.M., Melchior B., Schmid C.D., Ploix C.C. (2006). CNS immune privilege: Hiding in plain sight. Immunol. Rev..

[104] Qin L., Wu X., Block M.L., Liu Y., Breese G.R., Hong J.S., Knapp D.J., Crews F.T. (2007). Systemic LPS causes chronic neuroinflammation and progressive neurodegeneration. Glia.

[105] Zhang F., Jiang L. (2015). Neuroinflammation in Alzheimer’s disease. Neuropsychiatr. Dis. Treat..

[106] Heppner F.L., Ransohoff R.M., Becher B. (2015). Immune attack: The role of inflammation in Alzheimer disease. Nat. Rev. Neurosci..

[107] McGeer E.G., McGeer P.L. (2001). Innate immunity in Alzheimer’s disease: A model for local inflammatory reactions. Mol. Interv..

[108] Steinman L. (2013). Inflammatory cytokines at the summits of pathological signal cascades in brain diseases. Sci. Signal..

[109] Lustbader J.W., Cirilli M., Lin C., Xu H.W., Takuma K., Wang N., Caspersen C., Chen X., Pollak S., Chaney M. (2004). ABAD directly links Abeta to mitochondrial toxicity in Alzheimer’s disease. Science.

[110] Caspersen C., Wang N., Yao J., Sosunov A., Chen X., Lustbader J.W., Xu H.W., Stern D., McKhann G., Yan S.D. (2005). Mitochondrial Abeta: A potential focal point for neuronal metabolic dysfunction in Alzheimer’s disease. FASEB J..

[111] Gibson G.E., Karuppagounder S.S., Shi Q. (2008). Oxidant-induced changes in mitochondria and calcium dynamics in the pathophysiology of Alzheimer’s disease. Ann. N. Y. Acad. Sci..

[112] Coskun P.E., Wyrembak J., Derbereva O., Melkonian G., Doran E., Lott I.T., Head E., Cotman C.W, Wallace D.C. (2010). Systemic mitochondrial dysfunction and the etiology of Alzheimer’s disease and down syndrome dementia. J. Alzheimers Dis..

[113] Lubik N. (2008). Opening the “green pharmacy”. Environ. Sci. Technol..

[114] Rastogi T., Leder C., Kümmerer K. (2015). Re-Designing of Existing Pharmaceuticals for Environmental Biodegradability: A Tiered Approach with β-Blocker Propranolol as an Example. Environ. Sci. Technol..

[115] Larsen T.A., Hoffman S., Luthi C., Truffer B., Maurer M. (2016). Emerging solutions to the water challenges of an urbanizing world. Science.

